# Validation of associations for female fertility traits in Nordic Holstein, Nordic Red and Jersey dairy cattle

**DOI:** 10.1186/1471-2156-15-8

**Published:** 2014-01-15

**Authors:** Johanna K Höglund, Goutam Sahana, Bernt Guldbrandtsen, Mogens S Lund

**Affiliations:** 1 Faculty of Science and Technology, Department of Molecular Biology and Genetics, Aarhus University, P.O. Box 50, DK-8830 Tjele, Denmark; 2 VikingGenetics, Ebeltoftvej 16, Assentoft, DK-8960 Randers SØ, Denmark; 3 Department of Animal Breeding and Genetics, Swedish University of Agricultural Sciences, P.O. Box 7070, 750 07 Uppsala Sweden

**Keywords:** Female fertility, GWAS, Validation

## Abstract

**Background:**

The results obtained from genome-wide association studies (GWAS) often show pronounced disagreements. Validation of association studies is therefore desired before marker information is incorporated in selection decisions. A reliable way to confirm a discovered association between genetic markers and phenotypes is to validate the results in different populations. Therefore, the objective of this study was to validate single nucleotide polymorphism (SNP) marker associations to female fertility traits identified in the Nordic Holstein (NH) cattle population in the Nordic Red (NR) and Jersey (JER) cattle breeds. In the present study, we used data from 3,475 NH sires which were genotyped with the BovineSNP50 Beadchip to discover associations between SNP markers and eight female fertility-related traits. The significant SNP markers were then tested in NR and JER cattle.

**Results:**

A total of 4,474 significant associations between SNP markers and eight female fertility traits were detected in NH cattle. These significant associations were then validated in the NR (4,998 sires) and JER (1,225 sires) dairy cattle populations. We were able to validate 836 of the SNPs discovered in NH cattle in the NR population, as well as 686 SNPs in the JER population. 152 SNPs could be confirmed in both the NR and JER populations.

**Conclusions:**

The present study presents strong evidence for association of SNPs with fertility traits across three cattle breeds. We provide strong evidence that SNPs for many fertility traits are concentrated at certain areas on the genome (BTA1, BTA4, BTA7, BTA9, BTA11 and BTA13), and these areas would be highly suitable for further study in order to identify candidate genes for female fertility traits in dairy cattle.

## Background

There is often pronounced disagreement between different populations in the results obtained from genome-wide association studies (GWAS). Validation of association studies is therefore desired before marker information is incorporated in selection decisions, or before large sums are invested into identification of causal factors. The probability of observing spurious associations between a particular trait and SNPs in multiple populations by chance is small, particularly if significant associations are confirmed in two or more validation populations or breeds [1,2].

As a result of the widespread use of artificial insemination (AI), effective dairy cattle population sizes are relatively small. This has had an effect on patterns of linkage disequilibrium (LD) in dairy cattle breeds. For example, The Bovine Hapmap Consortium [3] reported low but non-zero levels of LD of up to 1,000 kb in several dairy breeds, in contrast to humans in which LD is found only up to tens of kb [4]. Because GWAS exploits LD, it should be possible to find significant associations in dairy cattle with markers positioned every 100 kb or so [5]. On the other hand, the level of LD also limits the precision of the QTL location, as SNPs at longer distances will exhibit association due to extended LD with causal mutation. This extended LD is not expected to exist across breeds, therefore across-breed validation of associations may help to narrow down the QTL interval.

Cattle breeding organizations in Denmark, Sweden and Finland participate in a joint breeding value estimation system, known as the Nordic Cattle Breeding Evaluation (NAV). Trait definitions are standardized across the countries and collected data is housed in a centralized database. The increased total number of records in pooled datasets allows for reliable detection of associations for traits with low heritability. NAV undertakes breeding value estimation for three breeds: Nordic Holstein (NH), Nordic Red (NR) and Jersey (JER). The standardized phenotype recording for female fertility traits across these three breeds provides unique data for discovering and validating female fertility associations. The objective of this study was to detect associations for female fertility traits in the NH population and to validate the discovered associations in the NR and JER breeds.

## Methods

DNA was extracted from semen samples from sires for genotyping in a previous project, so no ethical approval was required for this study.

### Animals and phenotype data

Data from three Nordic cattle breeds was used in this study. A total of 3,475 NH sires from Denmark, Sweden and Finland with official breeding values for female fertility traits were used to discover associations. Data from NR and JER cattle was used for population validation. There were 4,998 and 1,225 individuals with official breeding values in the NR and JER breeds, respectively. The numbers of animals with official breeding values for each of the eight fertility-related traits for the three breeds studied are presented in Table 1. Single-trait breeding values (STBV) were predicted for each animal using first to third parity data with best linear unbiased prediction procedures and a sire model where sires were treated as unrelated. This means that the STBV of a sire is predicted from its daughters’ information only. These STBVs were generated specifically for QTL mapping studies by the Nordic Cattle Genetic Evaluation company (http://www.nordicebv.info), and were adjusted for the same systematic environmental effects as in official routine evaluations. The traits evaluated included: number of inseminations per conception (AIS), 56-day non-return rate (NRR), days from first to last insemination (IFL), and the length in days of the interval from calving to first insemination (ICF). The Nordic female fertility index (FTI) is a compound index, based on data collected from Denmark (since 1985), Sweden (since 1982) and Finland (since 1994). The traits included in the FTI were: AIS in cows and heifers; ICF in cows; IFL in cows and heifers; NRR in cows and heifers; and heat strength (HS) in cows and heifers. FTI is meant to reflect the ease with which a cow or heifer was able to conceive. The economic value of HS and NRR were set to zero and therefore had no weight in the FTI. With the exception of ICF, STBVs from the national evaluation were available for both heifers, suffixed H, and cows, suffixed C. For details regarding the phenotypes recorded and models used in routine breeding value prediction, see http://www.nordicebv.info.

**Table 1:** Number of records analyzed for each female fertility trait in three cattle breeds

**Trait**^**1**^	**Nordic Holstein**	**Nordic Red**	**Jersey**
AISC	3,380	4,629	1,127
AISH	3,465	4,436	1,147
ICF	3,384	4,613	1,132
IFLC	3,394	4,637	1,135
IFLH	3,464	4,432	1,145
NRRC	3,394	4,636	1,133
NRRH	3,465	4,437	1,148
FTI	3,475	4,719	1,156

^1^AIS: number of inseminations per conception, NRR: 56-day non-return rate, IFL: days from first to last insemination, ICF: length in days of the interval from calving to first insemination, FTI: fertility index. C and H denote whether the trait has been measured in cows or heifers, respectively.

### SNP chip and genotyping

All animal samples were genotyped using the BovineSNP50 beadchip (Illumina, San Diego, CA), which assayed 54,001 SNP markers with a median interval of 37 kb between SNPs [6]. For these experiments, genomic DNA was extracted from whole blood or semen. The Illumina® Infinium II multi-sample assay protocol was followed to prepare SNP chips for scanning using the iScan imaging system. Analysis was performed using Beadstudio software (version 3.1). The quality parameters used for selection of SNPs in the study were minimum call rates of 85% for individuals and 95% for loci. Marker loci with minor allele frequencies (MAFs) below 5% were excluded. The minimum acceptable GC score [7] was 0.60 for individual typing, and individuals with average GC scores below 0.65 were excluded. SNP positions within a chromosome were designated according to the *Bos taurus* genome UMD3.1 assembly [8]. After filtering for poor quality data, a total of 38,545 SNPs on 29 bovine autosomes (BTAs) were used to determine associations in the NH population. NR and JER individuals were genotyped with the same BovineSNP50 beadchip and the quality control standards used for the NH population were used. However, only SNPs that showed genome-wide significant associations in the discovery population (in NH) and also passed the within-breed quality control criteria for genotypes were tested in the two validation populations.

### Statistical analysis

A SNP-by-SNP analysis was carried out in which each SNP was tested in turn for association with phenotypes. The following linear mixed model was used to estimate SNP effects in the NH population:

$$y_{\mathrm{ij}}=m+bx_{\mathrm{ij}}+s_{i}+e_{\mathrm{ij}}$$

Where y_*ij*_ was the single trait estimated breeding value of individual *j*, belonging to the half-sib (sire) family i, μ was the general mean, b is the allelic substitution effect, x_ij_ was the number of copies of an allele (with an arbitrary labeling) of the SNP count in the individual *j* (corresponding to 0, 1, or 2 copies), and s_*i*_ was the random effect of the i-th half-sib family and assumed to have covariances according to the relationship among sires. For example, s = {si} is normally distributed $N\left(0,\sigma_{g}^{2}A_{s}\right)$, where $\sigma_{g}^{2}\;$ is the polygenic genetic variance and *A*_*s*_ is the additive relationship matrix among the sires derived from the pedigree, and e_*ij*_ was a random residual of individual *i,* assumed to follow a normal distribution with mean zero and unknown variance. Testing was done using a *t*-test against a null hypothesis of H_0_: b = 0. The significance threshold was determined using a Bonferroni correction (within trait using 38,545 SNP markers). The genome-wide significance threshold was 1.3×10^-6^, and was calculated by dividing the nominal significance threshold of 0.05 by the total number of SNPs included in the analysis. The analysis were carried out using DMU software (http://www.dmu.agrsci.dk)

### Validation of associations

The genome-wide significant SNPs were selected from the NH discovery population. These SNPs were then tested in the NR and JER populations using the same model as that described above for NH. For the NR population, an additional fixed effect of breed was fitted as individuals in this population are derived from Danish Red, Swedish Red and White, and Finnish Ayrshire breeds. A stringent threshold for validation study will result in a few validated associations. As we have used a stringent criterion in the discovery population, a threshold of P < 0.05 was used in the validation populations. Therefore, if a SNP showed a genome-wide significant association for a trait in the NH population, and also showed a significant association (P < 0.05) for the same trait in the NR and JER populations, the association was considered validated. Using one validation population, the risk of falsely validating of an effect is 0.05. The risk of falsely validating in exactly one of the two validation populations is 2*0.05 – (0.05)^2^ = 0.0975. Finally, the risk of simultaneous falsely validating in two populations is (0.05)^2^ = 0.0025.

## Results and discussion

The present association study identified a total of 4,474 genome-wide significant SNPs associated with eight female fertility traits in the NH population (Additional file 1: Table S1). The most significant SNPs identified were associated with the FTI (973), IFLC (839), NRRC (758) and ICF (715) traits (Table 2). In general, more SNPs were detected for cow traits (AISC, IFLC, NRRC) than for heifer traits (AISH, IFLH, NRRH). The most significant SNPs were detected on BTA1 (548), BTA3 (368), BTA9 (333), BTA8 (288), BTA13 (279), BTA11 (251), BTA4 (219) and BTA26 (201). The lowest number of significant SNPs was detected on BTA27 (26) and BTA28 (21).

**Table 2:** Number of SNP found to be genome-wide significant in Nordic Holstein (NH) discovery population and validated at nominal significant level (P < 0.05) in Nordic Red (NR) and Jersey (JER)

**Trait**^**1**^	**No. of genome-wide significant associations in NH**	**Validated in NR**	**Validated in JR**	**Validated in both NR and JR**	**Chromosome where the validated SNPs are located**
AISC	490	81	79	23	1,2,3,4,5,6,7,8,9,10,11,13,14, 16,18,19,20,22,23,24,25,26,28
AISH	117	14	22	2	1,2,5,8,9,11,14,17,19,24,26,29
ICF	715	131	125	28	1,2,3,4,5,6,7,8,9,10,11,12,13, 14,15,16,17,18,19,20,21,22,23,24,26
IFLC	839	163	111	18	1,2,3,4,5,6,7,8,9,10,11,12,13, 14,15,16,17,18,19,20,21,22,23,24,25,26,27,28
IFLH	148	33	28	9	1,2,4,5,9,11,14,15,17,1,19,20, 22,26,27,29
NRRC	758	124	85	25	1,3,4,5,6,7,8,9,10,11,12,13,14,15,16,17,18,19,21,22,23,24,25,26,28,29
NRRH	434	68	66	13	1,2,3,4,5,6,7,8,9,10,11,12,13, 14,15,17,19,23,26,27,28,29
FTI	973	222	170	34	1,2,3,4,5,6,7,8,9,10,11,12,13, 14,15,16,17,18,19,20,21,23,24,25,26,27,28,29

^1^AIS: number of inseminations per conception, NRR: 56-day non-return rate, IFL: days from first to last insemination, ICF: length in days of the interval from calving to first insemination, FTI: fertility index. C and H denote whether the trait has been measured in cows or heifers, respectively.

According to previous studies [2,9] the only reliable way to confirm associations is to validate the results in different populations. We were able to validate 836 SNPs in the NR population and 686 SNPs in the JER population. Out of these 1,522 validated SNPs, 152 could be confirmed in both the NR and JER populations (Table 2 and Additional file 2: Table S2). The FTI trait had the highest number (34) of validated associations across the three breeds, followed by ICF (28). We observed only a few validations across the three breeds for the AISH (2) and IFLH (9) traits. In this study we used distantly related breeds for validation of associations. Therefore, the main reason for failure to validate many associations could be due to the fact that some NH associations were not segregating in the JER and NR breeds. The power of detecting associations also depends on minor allele frequencies and the genetic background. Therefore, the probability of detecting a QTL in three independent populations is reduced drastically even if the power to detect the QTL is high in one population. For example, if the power to detect a QTL is 80% in all three populations individually, the joint probability of this QTL being detected in all three populations is only 51%.

Pryce et al. [10] carried out a study to validate QTLs affecting female fertility (pregnancy within the first 42 days of mating). The Holstein cattle were used as the discovery population, while a new sample from Holstein and another Jersey population were used to validate associations. However, they could not validate any SNPs associated with female fertility across breeds. This exposes the challenges associated with mapping and validating associations related to female fertility. In contrast, we were able to validate a large number of associations due to the availability of large samples from three breeds and due to the uniform phenotype definitions across these three breeds. The correlation between the absolute values of the validated SNP effect sizes was higher between the NH and NR (0.18) populations compared to that of the NH and JER (0.10) populations. The correlation between the absolute values of the validated SNP effect sizes between JER and NR was 0.02. The correlations between the absolute effect sizes for the validated SNP-by-trait associations were low. This illustrates that marker associations between SNPs and Quantitative Trait Nucleotides (QTNs) are generally not well preserved between breeds. This may reflect a closer relationship between NH and NR than between NH and JER, in part due to the import of Holstein genetics to some portion of the NR breed. The difference in correlations could also be due to a more reliable estimate of SNP effects in NH and NR due to the larger sample sizes compared to those for the JER population. Pryce et al. [10] also reported a very low correlation (0.04) between absolute effect sizes between NH and JER for fertility.

The results from the present study were also compared to previous studies. However, direct comparison between the results obtained in this study and those from previous studies is hindered by the fact that locations from linkage analyses are mostly given in centimorgans (cM) and do not necessarily reflect the same physical location on the genome as the results derived from different genome assemblies. There are also differences in trait definitions across countries, which makes comparison difficult.

### Fertility QTLs detected

Many significant SNP markers were detected for female fertility traits in this study (Additional file 1: Table S1). As the aim of our study was to validate significant SNP markers in other breeds, we decided to focus on those chromosomes that harbored validated, significant SNP markers in all three breeds. The chromosomes harboring multiple QTLs, which were also validated in the NR and JER populations, were BTA1, BTA4, BTA7, BTA9, BTA11, and BTA13. These chromosomes are discussed in detail below.

### BTA1

On BTA1 several SNPs were detected with association to seven of the eight fertility traits analyzed (AISC, ICF, IFLC, IFLH, NRRC, NRRH, and FTI). For all of these traits, SNPs were confirmed in both the NR and JER populations (Additional file 2: Table S2). The confirmed SNP markers were concentrated in the range of 77 Mb to 100 Mb for AISC, IFLC, NRRC, NRRH, and FTI, whereas ICF and IFLH had a confirmed SNP in the range of 55 Mb to 60 Mb. Interestingly, AISC showed 6 confirmed SNP markers (BTA-36624-no-rs, UA-IFASA-6360, Hapmap52416-rs29016842, BTA-13139-rs29018103, BTB-01195902 and BTA-108728-no-rs) in the area between 77 Mb and 100 Mb. Furthermore, the AISH, IFLH and NRRH traits have their highest peak location in the same area (Additional file 1: Table S1). The proximity of the concentrated association peak suggests that the associations for these traits may reflect the same underlying causative mutations. Previously, Sahana et al. [11] detected SNP markers associated with FTI within the same area as the SNPs associated with FTI in our study. In fact, SNP rs29019866 was found to be significant in both studies. However, a portion of the NH data used in our study was also used by Sahana et al. [11]. Ben-Jemaa et al. [12] detected QTLs for non-return rate within the marker brackets INRA073 (78.9 Mb) and BM1824 (132.5 Mb), which is in the vicinity of the SNPs associated with FTI in our study. Significant SNP markers for AISH were also detected in the Finnish Ayrshire breed at 88 Mb [13], however part of the NR validation population used in the present study was also used in this previous study. In addition, a QTL was detected at marker INRA073 (78.9 Mb) in French dairy cattle for the trait of success or failure of each insemination [14]. Taken together, these findings strongly imply that the areas between 55 Mb to 60 Mb and 77 Mb to 100 Mb on BTA1 affect fertility in dairy cattle.

### BTA4

BTA4 harbored several SNPs with association to five fertility traits (AISC, FTI, ICF, IFLC, NRRC), which were confirmed in both the NR and JER populations (Additional file 2: Table S2). The SNPs associated with AISC, FTI, and IFLC showed overlap in the area of 50 Mb, indicating that the same genes may play a role in the regulation of these traits. The SNPs detected for ICF (40 Mb) and NRRC (69 – 80 Mb) were on a different location on the chromosome. In the vicinity of the associated SNP markers for ICF, a previous study [15] detected a QTL associated with IFLC. In addition, SNPs associated with fertility treatments, AISC, and IFLC have been detected on this chromosome in the vicinity of the regions found in this study [11]. A SNP association for NRRH was previously detected at 35 Mb in the Finnish Ayrshire population [13]. In addition, previous work detected significant SNP markers linked to *SCRN1* in the area of 69 Mb [16], and a QTL for daughter fertility on BTA4 was linked to the gene *ADCY1*[17]. In the present study, we detected SNPs associated with four different fertility traits (FTI, ICF, IFLC and NRRC) near the *ADCY1*(76,9 Mb) gene. The fact that the SNPs of these four different traits overlap indicates that these traits can be regulated by the same gene(s). *Leptin* (93,2 Mb), which is also located on BTA4, is also associated with fertility [18,19]. Even though validated SNPs were detected in the vicinity of the *leptin* gene, SNP markers within the *leptin* gene [18] were not present on the SNP array used in this study.

### BTA7

On BTA 7 SNP associated with FTI, IFLC, and AISC were overlapping. The same SNP markers for each trait could be validated in NR and JER (ARS-BFGL-NGS-81749, BFGL-NGS-119353, BTB-01207097). The validated SNP markers occurred around 40 Mb and 90 Mb. These findings strongly suggest that this area of the genome influences female fertility in dairy cattle. A previous study [11] detected a SNP marker for IFLC, and the same SNP marker (ss117968986) was associated with AISC in our study. In addition, a QTL for female fertility was detected close to marker INRA053 on BTA7 [14]. However, the confidence interval was large and therefore it is difficult to tell whether these QTLs overlap with the associations found in our study.

### BTA9

SNPs associated with eight fertility traits (IFLC, IFLH, ICF, NRRC, NRRH, AISC, AISH, and FTI) were detected on BTA9. SNPs for IFLC, NRRC and FTI were validated for all three breeds in the area of 90 Mb to 100 Mb. Furthermore, SNPs for AISC, IFLC, FTI, and NRRC were validated in the range of 55 Mb to 70 Mb, whereas confirmed SNPs associated with ICF were located at 25 Mb and 30 Mb. A previous study [11] detected SNPs associated with AISC, FTI and fertility treatments on BTA9, however none of these SNPs were among the validated SNPs detected in our study. Two separate studies [20,21] detected a QTL for non-return rate near the marker TGLA73 (76.7 Mb), which coincides with the BTA9 region containing SNPs detected for AISC, FTI, IFLC and NRRC in the present study. Combined linkage and linkage disequilibrium analysis were used to fine-map this QTL to 27 cM [22], and a QTL for heat intensity was detected near the marker BMS817 (39.3 Mb) [21], which coincides with the QTLs we detected for AISH, AISC, FTI, ICF, IFLC, IFLH, NRRC, and NRRH.

### BTA11

Validated SNPs associated with all eight fertility traits (AISH, AISC, ICF, NRRH, NRRC, IFLC, IFLH, and FTI) were detected on BTA11. Markers for IFLH, IFLC and FTI centered in the area of 80 Mb to 85 Mb, while markers for NRRC and ICF were located in the area of 60 Mb. SNPs were also validated in the area between 0 Mb and 33 Mb for NRRC, NRRH, and FTI. Interestingly, the NRRC and ICF traits showed overlap on two different regions of the genome with strong association in the NH population. However, these markers could not be validated in either the NR or JER populations. Even though BTA11 shows many significant SNP markers for multiple traits, only a few of them could be validated in the NR and JER breeds (ICF(1), FTI(2), IFLC (1)). A previous study [15] detected a QTL for ICF in the area of 84 Mb to 90 Mb, and three significant SNP markers for daughter fertility were previously detected on BTA11 [17]. One of these SNP markers was detected in the area of the validated IFLC SNPs detected in this study (29.0 Mb) and one was detected in the vicinity of the SNPs we detected and validated for NRRC and ICF. These two SNP markers were linked to the *PPM1B*(26,4 Mb) and *SLC1A4* (63.3 Mb)genes, respectively.

### BTA13

We identified 117 significant SNP markers for ICF on BTA13. Of the 117 significant SNP markers, 5 could be validated in both the NR and JER populations. Four SNPs were validated in the area from 38 Mb-45 Mb, while one SNP was validated around 65 Mb. In a previous study, a QTL for ICF was detected between markers BL1071 (71.9 Mb) and AGLA232 (77.6 Mb) by using linkage analysis [15]. In addition, Sahana et al. [11] detected SNP markers for ICF on BTA13. Seven of these SNP markers were also significant in our study, however none of these markers were validated in the NR or JER populations. Schulman et al. [13] also detected a significant association with ICF at 18.1 Mb on BTA13, and Huang et al. [23] detected a QTL for fertilization rate (77.0 Mb and 83.7 Mb) and blastocyst rate (1.8 Mb) on this chromosome. These findings make BTA13 particularly interesting for further analysis to identify the genes underlying genetic variation in ICF.

### Overlap between cow and heifer fertility associations

The genetic correlation between cow and heifer fertility traits varies in the literature but is generally low [24,25]. This indicates that the genes involved in the fertility traits of a heifer are different from those of a lactating cow. This is reflected in the results of this association study, in which little QTL overlap between cow and heifer fertility traits was found. These results are in agreement with those of a previous linkage study in which we detected little QTL overlap between cow and heifer traits [15].

### Association analysis model

Complex familial relationships are the primary confounding factor in dairy cattle GWAS studies [26]. When familial relatedness exists, the linear mixed model that accounts for the relatedness among all individuals is a useful approach in reducing the false positive rate [27]. In these analyses, a polygenic random effect term is included to represent the family structure. However, this approach is computationally challenging for large datasets. A previous study [28] has shown that a ‘compressed’ model in which individuals are clustered into groups can maintain a power similar to the linear mixed model of Yu et al. [27]. At the same time, the compressed model markedly reduces computing time. In these analyses, we have fitted a pedigree-based sire model, which considers the relationships among the grandsires to account for the relationship among the half-sib families.

We observed that the distribution of P-values had an intermediate to large genome-wide inflation. The lambda-values that reflect the genome-wide inflation of the test statistics [29] ranged from 2.6 to 4.7 for different traits. There are several potential explanations for this. First, the linkage disequilibrium in the NH population is extended to a very long range and the effective NH population size is around 50 [30]. Therefore, in a single marker analysis, all the SNPs in linkage disequilibrium with QTNs will have an effect on the analyses. Therefore, for each causal mutation 100 s of SNPs could show associations with traits. Second, the fertility traits analyzed in the present study are under intense directional artificial selection. This can result in genome-wide inflation of P-values. Such inflation will differ among traits depending on selection pressure. The highest lambda-value of 4.7 was observed for FTI, which is a combined index of fertility traits and is in the breeding goal. We have not corrected the test statistics using lambda-values. The inflation observed in this analysis is more likely due to high LD and artificial selection of the traits rather than to familial sub-structure, and the suitability of controlling the inflation through methods like genomic control in such situations [29] needs to be studied.

## Conclusions

Validation of association studies is important before marker information is incorporated in selection decisions or before large sums is invested into identification of causal factors. The present study presents strong evidence for association of SNPs with fertility traits across three cattle breeds. We provide strong evidence that SNPs for many fertility traits are concentrated at certain areas on the genome (BTA1, BTA4, BTA7, BTA9, BTA11 and BTA13), and these areas would be highly suitable for further study in order to identify candidate genes for female fertility traits in dairy cattle.

## Availability of Data

No new SNPs were discovered in this manuscript. All DNA sequences used were taken from a publicly available assembly. The assembly is available for download (ftp://ftp.ensembl.org/pub/release-73/fasta/bos_taurus/dna).

## Competing interests

The authors declare that they have no competing interests.

## Authors’ contributions

Conceived and designed the experiment: JKH, GS, BG, MSL. Analysed the data: JKH, GS. Contributed reagents/materials/analysis tools: MSL, BG, GS. Wrote the paper: JKH. All authors read and approved the final manuscript.

## Supplementary Material

Additional file 1: Table S1 Significant SNPs for female fertility traits in Danish Holstein cattle. Click here for file

Additional file 2: Table S2 Significant SNPs for female fertility traits in Danish Holstein cattle which are confirmed in Danish Jersey and Nordic red cattle. Click here for file

## References

[B1] Karlsson EK Baranowska I Wade CM Salmon Hillbertz NHC Zody MC Efficient mapping of Mendelian traits in dogs through genomewide association Nature Genet 2007 39 1321 1328 10.1038/ng.2007.10 17906626

[B2] Visscher PM Sizing up human height variation Nature Genet 2008 40 489 490 10.1038/ng0508-489 18443579

[B3] Bovine Hapmap Consortium Genome-wide survey of SNP variation uncovers the genetic structure of cattle breeds Science 2009 324 528 532 19390050 10.1126/science.1167936 PMC2735092

[B4] Tenesa A Navarro P Hayes BJ Duffy DL Clarke GM Recent human effective population size estimated from linkage disequilibrium Genome Res 2007 17 520 526 10.1101/gr.6023607 17351134 PMC1832099

[B5] de Roos APW Hayes BJ Spelman RJ Goddard ME Linkage Disequilibrium and Persistence of Phase in Holstein–Friesian, Jersey and Angus Cattle Genetics 2008 179 1503 1512 10.1534/genetics.107.084301 18622038 PMC2475750

[B6] Matukumalli LK Lawley CT Schnabel RD Taylor JF Allan MF Development and characterization of a high density SNP genotyping assay for cattle PLoS ONE 2009 4 e5350 10.1371/journal.pone.0005350 19390634 PMC2669730

[B7] Teo YY Inouye M Small KS Gwilliam R Deloukas P A genotype calling algorithm for the Illumina BeadArray platform Bioinformatics 2007 23 2741 2746 10.1093/bioinformatics/btm443 17846035 PMC2666488

[B8] Zimin AV Delcher AL Florea L Kelley DR Schatz MC Puiu D Hanrahan F Pertea G Van Tassell CP Sonstegard TS Marçais G Roberts M Subramanian P Yorke JA Salzberg SL A whole-genome assembly of the domestic cow Bos taurus. Genome Biol 2009 10 R42 10.1186/gb-2009-10-4-r42 PMC2688933 19393038

[B9] Lander E Kruglyak L Genetic dissection of complex traits: guidelines for interpreting and reporting linkage results Nature Genet 1995 11 241 247 10.1038/ng1195-241 7581446

[B10] Pryce JE Bolormaa S Chamberlain AJ Bowman PJ Savin K A validated genome-wide association study in 2 dairy cattle breeds for milk production and fertility traits using variable length haplotypes J Dairy Sci 2010 93 3331 3345 10.3168/jds.2009-2893 20630249

[B11] Sahana G Guldbrandtsen B Bendixen C Lund MS Genome-wide association mapping for female fertility traits in Danish and Swedish Holstein cattle Anim Genet 2010 41 579 588 10.1111/j.1365-2052.2010.02064.x 20477799

[B12] Ben-Jemaa S Fritz S Guillaume F Druet T Denis C Eggen A Gautier M Detection of quantitative trait loci affecting non-return rate in French dairy cattle J Anim Breed Genet 2008 125 280 288 10.1111/j.1439-0388.2008.00744.x 18717969

[B13] Schulman NF Sahana G Iso-Touru T McKay SD Schnabel RD Mapping of fertility traits in Finnish Ayrshire by genome-wide association analysis Anim Genet 2011 42 263 269 10.1111/j.1365-2052.2010.02149.x 21554346

[B14] Boichard D Grohs C Bourgeois F Cerqueira F Faugeras R Detection of genes influencing economic traits in three French dairy cattle breeds Genet Sel Evol 2003 35 77 101 10.1186/1297-9686-35-1-77 12605852 PMC2732691

[B15] Höglund JK Guldbrandtsen B Su G Thomsen B Lund MS Genome scan detects quantitative trait loci affecting female fertility traits in Danish and Swedish Holstein cattle J Dairy Sci 2009 92 2136 2143 10.3168/jds.2008-1104 19389971

[B16] Pimentel EC Bauersachs S Tietze M Simianer H Tetens J Thaller G Reinhardt F Wolf E König S Exploration of relationships between production and fertility traits in dairy cattle via association studies of SNPs within candidate genes derived by expression profiling Anim Genet 2011 42 251 262 10.1111/j.1365-2052.2010.02148.x 21198698

[B17] Kolbehdari D Wang Z Grant JR Murdoch B Prasad A A whole-genome scan to map quantitative trait loci for conformation and functional traits in Canadian Holstein bulls J Dairy Sci 2008 91 2844 2856 10.3168/jds.2007-0585 18565942

[B18] Clempson AM Pollott GE Brickell JS Bourne NE Munce N Wathes DC Evidence that leptin genotype is associated with fertility, growth, and milk production in Holstein cows J Dairy Sci 2011 94 3618 3628 10.3168/jds.2010-3626 21700051

[B19] Chebel RC Santos JEP Association between leptin single nucleotide polymorphism and reproductive performance of lactating Holstein cows Anim Reprod Sci 2011 127 126 134 10.1016/j.anireprosci.2011.06.011 21917388

[B20] Schrooten C Bovenhuis H Coppieters W Van Arendonk JA Whole genome scan to detect quantitative trait loci for conformation and functional traits in dairy cattle J Dairy Sci 2000 83 795 806 10.3168/jds.S0022-0302(00)74942-3 10791796

[B21] Holmberg M Andersson-Eklund L Quantitative trait loci affecting fertility and calving traits in Swedish dairy cattle J Dairy Sci 2006 89 3664 3671 10.3168/jds.S0022-0302(06)72406-7 16899702

[B22] Holmberg M Sahana G Andersson-Eklund L Fine mapping of a quantitative trait locus on chromosome 9 affecting non-return rate in Swedish dairy cattle J Anim Breed Genet 2007 124 257 263 10.1111/j.1439-0388.2007.00669.x 17868077

[B23] Huang W Kirkpatrick BW Rosa GJ Khatib H A genome-wide association study using selective DNA pooling identifies candidate markers for fertility in Holstein cattle Anim Genet 2010 41 570 578 10.1111/j.1365-2052.2010.02046.x 20394602

[B24] Pedersen J Jensen J Evaluation of female fertility of Danish dairy sires Proceedings International workshop on workshop on genetic improvement of functional traits in cattle. January 1996 1996 12 Gembloux, Belgium: Interbull bulletin 72 77

[B25] Hansen LB Freeman AE Berger PJ Association of heifer fertility with cow fertility and yield in dairy cattle J Dairy Sci 1983 66 306 314 10.3168/jds.S0022-0302(83)81790-1 6833602

[B26] Sahana G Guldbrandtsen B Janss L Lund MS Comparison of association mapping methods in a complex pedigreed population Genet Epidemiol 2010 34 455 462 10.1002/gepi.20499 20568276

[B27] Yu J Pressoir G Briggs WH Vroh Bi I Yamasaki M Doebley JF McMullen MD Gaut BS Nielsen DM Holland JB Kresovich S Buckler ES A unified mixed-model method for association mapping that accounts for multiple levels of relatedness Nature Genet 2006 38 203 208 10.1038/ng1702 16380716

[B28] Zhang Z Ersoz E Lai CQ Todhunter RJ Tiwari HK Mixed linear model approach adapted for genome-wide association studies Nature Genet 2010 42 355 360 10.1038/ng.546 20208535 PMC2931336

[B29] Devlin B Roeder K Genomic control for association studies Biometrics 1999 55 997 1004 10.1111/j.0006-341X.1999.00997.x 11315092

[B30] Sørensen AC Sørensen MK Berg P Inbreeding in Danish Dairy cattle breeds J Dairy Sci 2005 88 1865 1872 10.3168/jds.S0022-0302(05)72861-7 15829680

